# Recommendations and future directions for supervised machine learning in psychiatry

**DOI:** 10.1038/s41398-019-0607-2

**Published:** 2019-10-22

**Authors:** Micah Cearns, Tim Hahn, Bernhard T. Baune

**Affiliations:** 1 Discipline of Psychiatry, School of Medicine, University of Adelaide, Adelaide, SA 5005 Australia; 2 Institute of Translational Psychiatry, University of Münster, 48149 Münster, Germany; 3 Department of Psychiatry, University of Münster, 48149 Münster, Germany; 4 Department of Psychiatry, Melbourne Medical School, The University of Melbourne, Parkville, VIC 3010 Australia; 5 The Florey Institute of Neuroscience and Mental Health, The University of Melbourne, Parkville, VIC 3010 Australia

**Keywords:** Scientific community, Predictive markers

## Abstract

Machine learning methods hold promise for personalized care in psychiatry, demonstrating the potential to tailor treatment decisions and stratify patients into clinically meaningful taxonomies. Subsequently, publication counts applying machine learning methods have risen, with different data modalities, mathematically distinct models, and samples of varying size being used to train and test models with the promise of clinical translation. Consequently, and in part due to the preliminary nature of such works, many studies have reported largely varying degrees of accuracy, raising concerns over systematic overestimation and methodological inconsistencies. Furthermore, a lack of procedural evaluation guidelines for non-expert medical professionals and funding bodies leaves many in the field with no means to systematically evaluate the claims, maturity, and clinical readiness of a project. Given the potential of machine learning methods to transform patient care, albeit, contingent on the rigor of employed methods and their dissemination, we deem it necessary to provide a review of current methods, recommendations, and future directions for applied machine learning in psychiatry. In this review we will cover issues of best practice for model training and evaluation, sources of systematic error and overestimation, model explainability vs. trust, the clinical implementation of AI systems, and finally, future directions for our field.

## Introduction

Accurate prediction of intervention response and illness trajectories remains an elusive problem for modern psychiatry, with contemporary practitioners still relying on a ‘wait and see’ approach for the treatment of psychiatric disorders^1^. This problem has likely arisen due to an interplay of biopsychosocial factors^2^ and statistical modeling decisions^3^. From a biopsychosocial perspective, the high degree of comorbidity between psychiatric conditions^4,5^, the lack of diagnostic biomarkers to delineate between disorders and illness trajectories^6,7^, the shared genetic origins of clinically disparate traits^8^, and the imprecision of symptom measures^9,10^ has likely contributed to the complexity and lack of accuracy in clinical decision making.

Methodologically, psychiatry has commonly focused on statistical inference over prediction^3,11^. Inferential statistics have afforded the testing of theory driven hypotheses, population inference, and the formulation of grounded theory and mechanism to better understand the aetiology of psychiatric traits^3,11^. However, both psychiatry and neuroscience have found themselves with significant translation problems. Even in the face of new discoveries and paradigm shifts in the understanding of disorders, the clinical practice of psychiatry and discovery of interventions that outperform placebo has been slow^12^.

Given this complexity, clinicians commonly assume diagnostic homogeneity, where all patients who present with e.g., symptoms of low mood, lack of energy, and negative thoughts are considered to have the same broad diagnosis of major depressive disorder (MDD)^1^. However, studies using machine learning methodologies (ML) have begun to identify subtypes of psychiatric disorders with differing symptomology^13^, illness trajectories^14,15^, and drug response profiles^16^. Adding another layer of complexity are the individual differences inherent in these disorders^17^. Ignoring this individuality and modeling at the group level fails to represent the heterogeneity of clinical populations^18^. Rather than assuming that all patients are accurately represented by measures of central tendency from case control studies, one solution is to utilize modeling techniques that can parse patient heterogeneity^19^.

ML models are capable of this task, by learning individual patient characteristics, they can make successive individual (i.e., single subject) predictions. For example, using a ML model trained on multisite data from the STAR*D consortium, Chekroud et al.^16^, were able to predict remission from MDD after a 12-week course of Citalopram therapy with an accuracy of 64.6%. The model was then externally validated in the escitalopram and escitalopram-bupropion treatment group of COMED, attaining accuracies 59.6% and 59.7%, respectively. Given a report of ~49.3% accuracy for clinician prognostication on the same outcome in the STAR*D cohort^16^, this is a clinically meaningful increase in prognostic certainty. This study is an exemplar of applied ML in psychiatry. The dataset had a large number of observations allowing for the learning of unique patient characteristics. Model selection was tailored to the available data and rigorously cross-validated across sites using pipeline architecture. It was multisite, affording geographic generalizability and a large clinical scope. Finally, code for the trained model was made available on request, allowing for transparency and dissemination of the studies methods.

Prior to and since this publication, many more ML works have been published using different data modalities, models, sample sizes, and most interestingly, have reported largely varying degrees of accuracy^20^. Given this variability, concerns have been raised questioning the veracity of findings in our field and hinted at sources of systematic overestimation^20,21^. Adding further complication, large multisite datasets are the exception, not the rule, commonly have more predictors than observations, are generally imbalanced, and have low signal to noise ratios. Given these circumstances, concerns have been raised that ML may face the same reproducibility crisis as that experienced by group level analyses in recent years^22–24^. However, when proper methodology has been employed, ML works have been shown to prognosticate significantly better than chance on unseen data^15^, generalize across data collection sites^14^, and outperform clinician prognostication^16,25^.

Given the potential of ML models to transform patient care, albeit, contingent on the rigor of employed methods, we deem it necessary to provide a best practice overview for applied ML in psychiatry. In this guide we will cover issues of best practice for model training and evaluation, sources of systematic error and overestimation, model explainability vs. trust, the clinical implementation of AI systems, and finally, future directions for our field.

### Model training and evaluation

#### Sample size and systematic overestimation

An array of model training and testing schemes exist in ML. The choice of which depends on the size of a dataset and a practitioner’s computational resources. This brings us to our first encounter with the question of sample size in psychiatric ML. How big should a dataset be before a practitioner decides to use an ML strategy? The ignorance of this question and application of ML to small datasets has given rise to an important concern: Particularly, ML studies using larger samples have commonly shown weaker performance than studies using smaller samples^26^. This observation has led to questions regarding the validity and reliability of preliminary small N ML studies in psychiatry^21^. To measure the degree of these effects, Neuhaus and Popescu^20,26^ collated studies across conditions; including schizophrenia (total observation *N* = 5563), MDD (*N* = 2042), and attention deficit hyperactivity disorder (ADHD, *N* = 8084), finding an inverse relationship between sample size and balanced accuracy (schizophrenia, *r* = −0.34, *p* = 0.012; MDD, *r* = −0.32, *p* = 0.053; and ADHD, *r* = −0.43, *p* = 0.044)^20^. As we would expect model performance to increase with more data, these findings suggest an underlying problem within our field.

One explanation proposed by Schnack and Kahn^21^ is that patient characteristics in smaller samples tend to be more homogenous. In the case of small N, participants may be more likely to be recruited from the same data collection site and of a similar age (for example, in the case of a university recruited convenience sample). In addition, stringent recruitment criterion may be easily met, resulting in a well-defined phenotype that is not truly representative of the parent population of interest. As sample size increases, the geographic, demographic, and phenotypic diversity of a sample will increase also, resulting in decreased model performance, yet, increased generalizability and model scope (see from proof-of-concept studies to clinical application below). Theoretically, future works may be able to circumvent this trade-off by subtyping patients into well-defined and clinically meaningful clusters, thus, models could be trained for specific patient subtypes, maintaining phenotypic homogeneity as sample size increases. These considerations underline the importance of further research into patient subtyping in conjunction with supervised ML methods. In addition to the issue of sample homogeneity, the sample size needed to train an ML model is also contingent on the strength of the underlying signal between input features and an outcome of interest, as well as the complexity of the underlying mapping function (the mathematical function used to derive a line or curve between datapoints). As these two factors can vary greatly between research questions and datasets, there can—in principle—be no general rule of thumb to estimate the sample size required for analyses. Beyond these sample size and heterogeneity constraints, the choice of cross-validation scheme also bears influence on the variability and bias of accuracy estimates. In the following sections, we will address the methods available for preliminary ML works and their common use cases.

#### Leave one out cross-validation

Due to sample size constraints, leave one out cross-validation (LOOCV) has become a popular strategy for model performance evaluation in applied psychiatric ML. With this strategy, a model is trained on all available observations minus one (*n* − 1). Following, the trained model is tested on the one held-out observation. This process is repeated until all available observations have been used for testing. Final model performance is then averaged across the held-out samples and an accuracy estimate is derived. However, previous work has demonstrated the variance properties of LOOCV, suggesting that although this method utilizes all available data, an appealing property given limited *N*, it consequently leads to unstable and biased estimates due to the high degree of correlation between samples^27^. Therefore, repeated random splits are preferred, showing less bias and variability^27,28^. Notwithstanding, further complications will arise when we conduct multiple transformations on our data and optimize a models hyperparameters. If we were to use LOOCV, yet, conduct transformations within the same cross-validation scheme, we may optimistically bias our model evaluations by using the same test set (*n* − 1) to select parameters and evaluate the model^29^. In this instance, we need a method that allows us to take advantage of all our data for both model selection and evaluation whilst avoiding any circularity bias. In these situations, each transformation needs to be completed in a nesting procedure.

#### Nested cross-validation

As the name implies, nested cross-validation allows for the nesting of multiple cross validations. First, an inner cross-validation loop is used to conduct data transformations and/or hyperparameter optimization. This loop is akin to a train/validation partition. Following, this loop is nested inside an outer cross-validation loop that assesses the transformed data and optimized model on different test sets to those used in the inner loop. This outer loop is akin to a validation/test partition and allows for the approximation of the selected models performance.

**Fig. 1:**
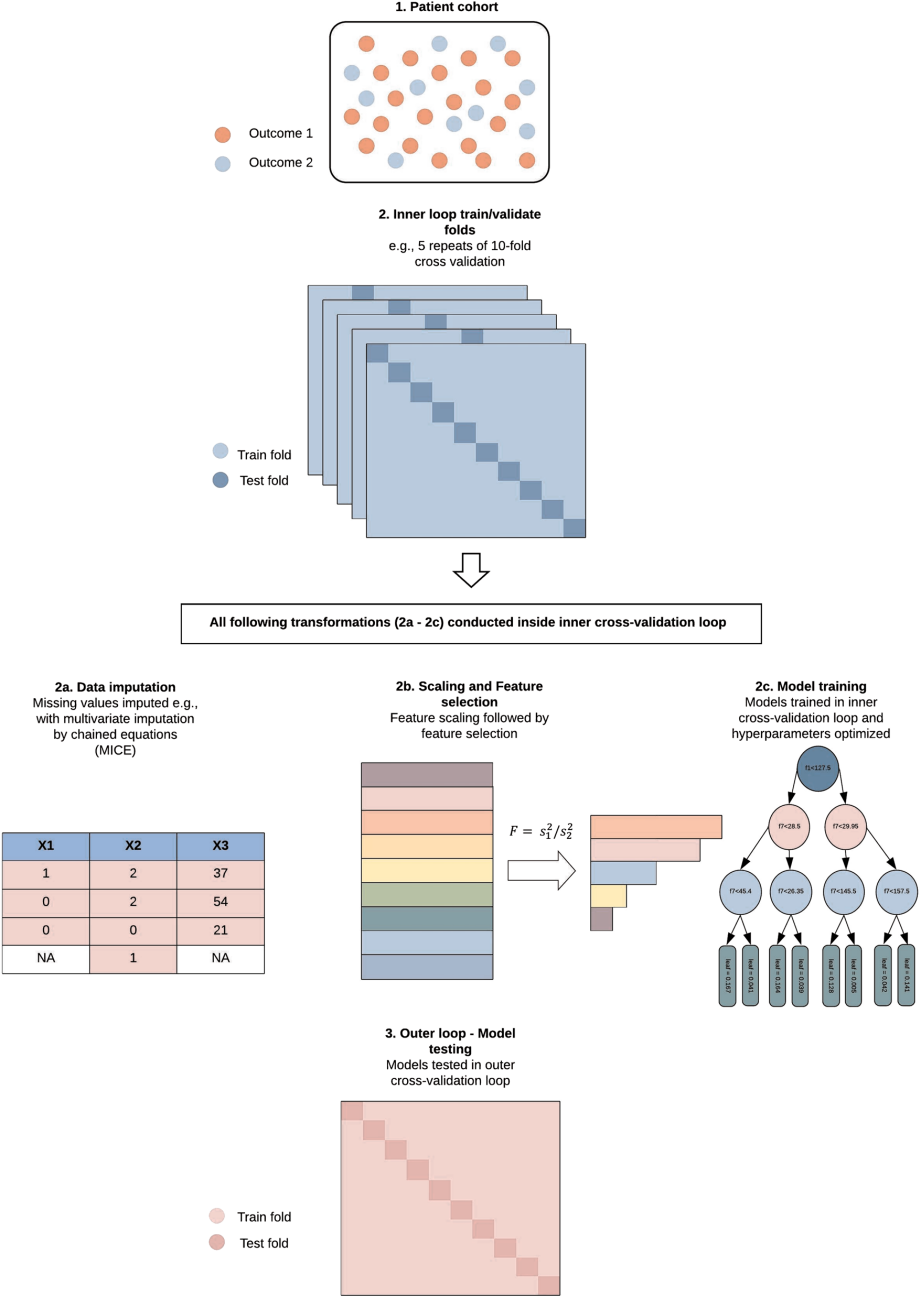
Visualization of a nested cross-validation scheme. All steps from 2a–2c should be conducted inside a pipeline, inside the inner cross-validation loop

Once this architecture is defined, the appropriate number of cross-validation iterations need to be set. Previous work by Kohavi has investigated this topic in detail^28^. For model selection, he found that 10-fold cross-validation best balanced the bias/variance trade-off. In addition, repeated runs of the 10-fold cycle were recommended to avoid opportune splits that may lead to overly optimistic estimates. Three to five repeats are commonly used. Finally, given the balance between computational cost and the bias/variance trade-off, it is common to use another 10-fold cycle in the outer cross-validation loop to assess the final model’s performance. See Fig. 1 for visualization of this structure. It is important to note that, no “optimal” number of repeats can be made as this will likely depend on the complexity of the model, the size and amount of signal in a sample, and the resulting stability of the final cross-validation estimates. If, for example, variance is high between cross-validation folds, more repeats should converge on a less variable mean estimate and reduce dependency on spurious data partitions. On the contrary, if the variance of estimates is low between folds, a high number of repeats will be of less use and increase computational cost. However, whether repeated runs actually achieve this intended variance reduction has been contested^30^. Nonetheless, in small samples it may decrease the effects of favorable cross-validation splits without compromising test set sizes in a k-fold scheme.

While this method provides a means to conduct multiple transformations and estimate parameters without overtly double dipping into a dataset, it is possible that a nested scheme may generate test sets that are overly similar to training sets, as well as similar training sets across cross-validation folds^29,31^. In theory, this could also lead to systematic overestimation. One alternative, in the case of small *N* is to keep data transformations and hyperparameter optimization to a minimum^27^. In the case of many features, only a few subsets could be tested in the inner loop whilst hyperparameters for the model could be left at default to decrease the risk of double dipping. For an example of this method in Python, see the following documentation^32^.

#### Train/test/validate

In the case of a sufficiently large sample, the problem of train/validate/test set overlap in a nested scheme can be avoided by using an entirely separate test partition to the train/validate partitions used for model selection. For example, if a balanced sample of *N* = 500 is available, *N* = 300 could be partitioned for training and validation, whilst *N* = 200 could be held out for final testing. In this case, both sample size over/under estimation and partition overlap could be minimized. Commonly, partitions will be stratified by the outcome label for prediction. Transformations and model parameters will be learnt in the train/validation partitions and analyzed in the remaining test partition. For deployment of this method in the Python programming language, see the following documentation^33^.

#### Leave group out cross-validation

What about when a sample is multisite, and each data collection site has its own unique characteristics? Further, what if outcome distributions vary across these sites? One site, a treatment center, may have primarily collected a convenience sample of patients, whilst another may have predominantly focused on controls. When we tune a models hyperparameters, how do we avoid tuning them directly to these differences that may proxy for disparities in outcome distributions or processing equipment? Here, the solution is leave-group out cross-validation, also known as Monte Carlo or leave site out cross-validation^34,35^. In this situation we need to assess whether a model trained on a particular data collection site, will generalize to other sites. To achieve this, we hold out samples according to a third-party array of integer groups that represent each site. These integer codes can then be used to encode site specific cross-validation folds and assess the generalizability of model performance. This approach ensures that all samples in the cross-validation folds come from sites that are not represented at all in the paired training folds. In situations where consortia/multisite data is used, this method is required. For deployment of this method in the Python programming language, see the following documentation^36^.

#### Data leakage

While the use of proper cross-validation schemes and minimum sample sizes helps reduce estimate variability and systematic overestimation, another overlooked source of error comes in the form of data leakage. The effects of leakage on estimates are profound yet appear to be rarely discussed in the psychiatric ML literature. Named one of the top 10 data mining mistakes^37^, leakage refers to the introduction of information about the outcome label (e.g., case/control) that would otherwise not be available to learn from. A trivial example of data leakage would be either selecting features or imputing missing values on an entire dataset before partitioning it into train/validate/test folds. Here, feature distribution information from the validate/test folds would be leaked into the train set, hyperparameters would be tuned to these distributions, and inevitably, test set outcomes would be predicted with a high degree of accuracy. For an in-depth appraisal of this problem and solutions, see Kaufman^37^.

This problem can be easily avoided through careful selection of features (only selecting features that would truly be available at time of analysis) and the partitioning of cross-validation folds prior to data transformations. However, as sample size is often constrained in psychiatric cohorts, as discussed above, automated k-fold and nested schemes are commonly used instead of a-priori train/validate/test partitions. As multiple k-fold runs are conducted, it is not as simple as learning transformations on a train partition and then predicting them into validation/test partitions. Here, data leakage risk increases substantially, yet can still be avoided through the use of pipeline architecture.

#### Pipeline architecture

A machine learning pipeline can be thought of as an object that sequentially chains together a list of transformers and a final estimator into one object. This sequential chaining has three advantages. First, a practitioner only has to call ‘fit’ and ‘predict’ once on a set of data, rather than at each transformation. Secondly, hyperparameters from each estimator can be tuned in unison. Finally, pipelines help avoid the leakage of statistics by guaranteeing that the same samples are used to train the transformers and the final classifier. It is easy to overlook the importance of this final step. As datasets are commonly repositioned for ML analysis, the a-priori pre-processing of data sets is common. As demonstrated above, if a transformation as simple as imputation is completed on a dataset in its entirety, this is enough to leak statistics and cause optimistic bias.

To demonstrate the correct use of pipeline architecture in the Python programming language and quantify the magnitude of data leakage effects, we have provided an example Python script in the references^38^. In this example, we randomly generate a balanced large P small N dataset containing 3000 features and 500 observations. To emulate the low signal to noise ratio commonly inherent in psychiatric cohorts, only 10 of the 3000 features are related to the binary outcome vector $y = \{ 1,0\}$. To demonstrate data leakage effects, we first mean center the dataset and select a subset of features using regularized logistic regression (LASSO)^39^ on the full dataset. Following, we train and test a linear SVM with default parameters using 10-fold cross-validation, attaining a test set AUC of 99.89. Next, we conduct the same procedure, now implementing each transformation within a sklearn pipeline to ensure the use of the same cross-validation folds over each transformation. Now, we attain a test AUC of 50.23. Here, we see that when a pipeline is not used, the model overfits to leaked statistics and appears to be a near perfect classifier. However, when we use a pipeline, conduct transformations in the same folds, and only learn from the true signal in the features, predictive accuracy is no better than chance.

Given that at current, the open sourcing of code for peer review is not requested by journals, and the ease of which these mistakes can be made by those newer to the field, it is possible that beyond just sample size effects, many of the highly optimistic studies ($\ge 90$%) may be due to data leakage. To counteract this problem, we recommend that journals require code reviews in the peer review process. In addition, the open sourcing of code should be encouraged. Beyond the aforementioned sample size estimation biases, such minor transgressions in code structure may go some way to explain the large degree of variability currently observed in the literature. Finally, permutation tests should be conducted regardless of cross-validation strategy deployed. This way, the null-distribution and statistical significance of a classifier can be obtained^40^.

### From proof-of-concept studies to clinical application

While consortia efforts and open sourced models are becoming more prevalent, publication numbers of studies at all points along the project maturity continuum continue to rise. Of utmost importance, ensuring methodological rigor and conceptual maturity of these publications is essential. However, beyond best practice recommendations like those provided above, the current lack of practical guidelines to systematically evaluate ML quality and maturity makes it difficult for researchers, stakeholders, journals, and funding agencies to objectively gauge the quality and current clinical utility of an ML model or publication. In addition, the lack of evaluation guidelines risks an overly optimistic or an unduly skeptical perception of findings—both in the scientific community and the public eye. Therefore, we propose a practical set of guidelines for the assessment of clinical utility and maturity for ML models in psychiatry. Based on the conceptual framework of *AI Transparency*^31^, we have derived a straightforward “checklist” with which to quantify a project’s maturity ranging from the initial proof-of-concept stage through to the clinical application stage. Specifically, the checklist comprises six categories in which an ML project is evaluated. In each category, scores range from “proof-of-concept stage” (0) to “ready for clinical application” (2).

The first category—generalization—refers to model performance in previously unseen data and has been outlined in detail above. It constitutes the most rudimentary performance measure of an ML model. While employing cross-validation techniques (score 0) avoids data leakage and provides a principled estimate of generalization performance, it is important to note that (nested) cross-validation should be used for initial model evaluation and hyperparameter optimization only. At current, the results of most machine learning studies in psychiatry might well have arisen from small test-sets as is typical for cross-validation. In contrast, using a large, independent test set (score 1) yields a more stable, reliable estimate of future performance. Finally, using a large, external test set (score 2), i.e., a test set to which the creators of the initial model did not have access at training time, and which was drawn independently from the training set, is optimal. To this end, online model repositories (e.g., www.photon-ai.com/repo) provide valuable infrastructure which greatly simplifies external validation in practice. Here, a research group can make available a trained model, and have it tested on independent data, providing an opportunity to assess the geographic, demographic, and phenotypic generalizability of a published model. In addition, incremental learning can be used to train certain ML models, allowing a research group to further train a pre-trained model with their own data, affording cross institution collaboration without the need for data sharing (for more information, see the partial fit method in sklearn^41^).

The second category—model scope—refers to the group of individuals about whom the model can make reasonable predictions. If a convenience sample (score 0) is used for training and testing, we cannot expect the model to perform well on different samples. If the sample is representative for a site or local subpopulation (score 1), we can expect it to perform as intended at this site without any guarantees with regard to other sites or subpopulations. Only testing on a representative sample of the target population (score 2) ensures reliable performance estimates that translate into clinical practice. From this point of view, the current use of exclusion criteria appears highly problematic. While perfectly reasonable if we seek to test hypotheses to gain insight into mechanisms or advance theory, excluding patients with e.g., certain comorbidities inevitably entails that our model’s utility for this patient group cannot be estimated, thereby severely hampering its applicability in clinical practice.

The third category—incremental utility—refers to the added value a machine learning model confers as compared to current practice^42^. While most studies do not assess incremental utility (score 0), showing higher efficiency or effectiveness with regard to the current state-of-the-art (score 1) is essential. If a model or project cannot show or does not intend to do this, little in the way of clinical translation can be expected. Thus, the essential goal should always be that a project reaches a stage in which it outperforms current state-of-the-art in real-life workflow (score 2). Although experimental approaches such as randomized controlled trials seem optimally suited to show incremental utility, they are rarely used in medical ML research today. This concept also highlights a misalignment of expectations with regards to model performance. A commonly held view is that a model needs to be highly accurate to be of clinical use. However, if a model can outperform its opportunity cost, that is, current state of the art clinical practice free of decision support systems, then patient utility can be maximized at scale. As previously mentioned, clinician rated accuracy to predict remission in the STAR-D cohort was ~49.3%, whilst that of a weak to moderately accurate AI model ranged between 59.6% to 64.6% accuracy. Whilst far from perfect, a clinically meaningful increase in prognostic certainty was attained. Therefore, the absolute accuracy of a classifier should not serve as an indicator of clinical utility, but the relative increase in prognostication compared to current state of the art practice.

**Fig. 2:**
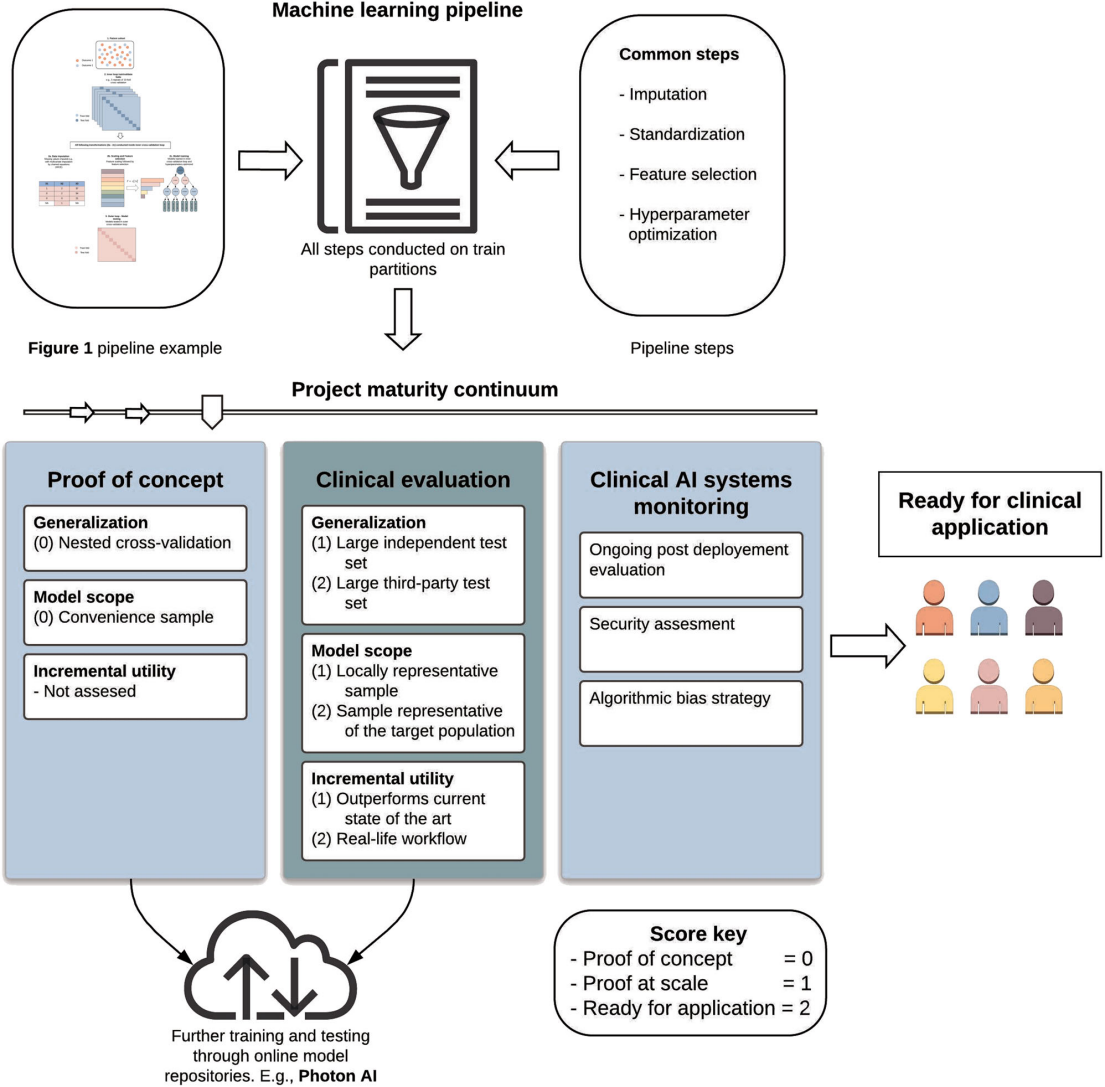
Illustration of the full best practice workflow from pipeline construction through to project maturity assessment. Dependent on the sample, crossvalidationscheme, and measurement of incremental utility compared to current clinical practice, a project can fall into 3 distinct phases of project maturity dictating its readiness for clinical use

Using this simple checklist, we can now evaluate a project or publication with regard to its position on the continuum spanning from “proof-of-concept stage” to “ready for clinical application”. Note that the three categories outlined above build upon each other in the sense that a model with bad generalization cannot have a broad model scope. Likewise, to show incremental utility, a model must generalize well and have reasonable model scope. Thus, insufficient scores in one category cannot be compensated by higher scores in any other category. See Fig. 2 for an illustration of this general workflow.

Once an ML model reaches the level we term “ready for clinical application”, other considerations regarding post-deployment evaluation, security, and algorithmic bias come into focus^31^. While often crucially important, their impact depends heavily on the context in which the model is used. If, for example, we could be sure that the model draws on measures of causal mechanisms, we can assume that the relationship will not change over time, rendering post-deployment evaluation less important. Also, an ML model deployed in a consumer application for smartphones might require much higher security (e.g., regarding adversarial attacks and data security, i.e., hacking of smartphone input data leading to erroneous model outputs and therapeutic decisions) than is necessary in a closed hardware-based device used in the clinic only. Finally, as ML models are not programmed, but trained, they will mimic systematic biases inherent in their training data. This potential algorithmic bias must be carefully investigated. While it can often be eradicated by retraining without the variables causing the bias, we need to be aware of a bias to be able to remove it. Thus, human involvement is crucial at this point (for an in-depth discussion, see ref. ^43^).

Importantly, these guidelines are intended to gauge ML model maturity with regard to clinical application only. It should explicitly not be applied to ML research projects developing methods and proof-of-concept studies. Judging those with the checklist outlined here would inevitably result in low scores and might therefore stifle even the most promising of methodological developments. It is this creativity and ingenuity, however, that allows the field to move at such breathtaking speed. If researchers claim to develop a clinically useful tool—as is done in the introductions and discussion sections of countless publications and funding proposals, even if nothing but classic statistical group inference or the simplest of ML models are planned—we can judge project planning, as well as subsequent results using this approach to delineate projects aiming for ML with high clinical utility from studies seeking insight into mechanisms or proof-of-concept studies. The ability of journals and funding agencies, as well as the general public to evaluate the aims and quality of research projects in this manner is crucial for all translational efforts in psychiatry.

### Understanding ML models

In the wake of ever-more powerful machine learning applications emerging across a range of industries, the question of explainability—i.e., understanding which (patterns of) variables lead to which model predictions—has increasingly come into focus. Identifying the (pattern of) variables driving predictions is of obvious scientific interest. Extracting these relevant variables in an ML study would provide theoretic insights similar to classical (usually univariate) statistical inference while retaining predictive performance. In addition, if the relevant variables could be extracted, equally well-performing models could be trained with only a small subset of variables, saving resources on all levels from data acquisition to processing and storage.

With regard to clinical evaluation as outlined in the previous section, quantifying the effect of variables can also be beneficial in at least three ways: First—with regard to model utility—identifying relevant variables might help to detect trivial and erroneous models. For example, Lapuschkin et al.^44^ used Layer-wise Relevance Propagation (LRP)^45^ to show that an ML model trained to detect certain objects in photographs (in this case horses) in fact used nothing but the text of a tag present on all images of horses in the training data. If this tag was inserted into other photographs, the images would be classified as horses independent of their actual content. In addition, identifying relevant variables might enable domain experts to judge whether the associations on which the model relies are likely to remain stable. Second—with regard to model fairness—knowledge of the relevant variables may help to identify algorithmic bias. If, for example, gender and age are identified as the most relevant variables in a model built to identify suitable job applicants, this bias could be explicitly addressed. Third—with regard to model security—identifying the relevant variables provides information on where the model could be most easily attacked. This, in turn, might help to immunize the model.

Given that (almost all) ML models are by no means “black boxes”, but apply a transparent and deterministic, albeit often rather complex rule to make predictions, a large number of algorithms aiming to help us understand this rule, i.e., to identify relevant variables, have been developed (for a general introduction, see ref. ^46^). While it is beyond the scope of this article to review the numerous families of such algorithms, they usually quantify the contribution of each individual variable used in a prediction. This can be done, for example, in a straightforward manner by systematically obscuring certain sets of variables in novel data and analyzing the resulting decrease in performance. Another group of approaches investigates the trained ML model itself. Examples of this include simple weight-maps for Support Vector Machines, decision tree-based importance scores in tree-based models, as well as more complex approaches, such as visualizing the process of layer-wise data transformation in neural networks, including LRP^45^, or more generally applicable approaches e.g., based on Game Theory^47^. While the wealth of research in this area has vastly increased the toolbox available for model interpretation in recent years, it also indicates that no complete solution has been found. In fact, different approaches may lead to different variables being identified.

However, the problems with ML model explainability run much deeper: On the one hand, all algorithms which identify variables driving model prediction require human interpretation. Given the multivariate nature of ML models, this renders such an interpretation extremely difficult for even the simplest of (linear) models. On the other hand, the insight into model utility, fairness, and security we can gain is not only very limited but can usually be accomplished much better by straightforward model evaluation. For example, while quantifying variable relevance might indicate that problematic variables (such as gender or age) are driving predictions, explicitly excluding these variables in the first place is much more effective. However, it is possible that seemingly bias free variables may be confounded even after the exclusion of problematic ones. Further analyses may be required to elucidate such hidden confounds prior to selecting variables for model consideration. Further work in this area is required and ongoing.

While we believe that complete transparency regarding the previously outlined checklist to be essential and facilitated by practices such as variable assessment for bias and the sharing of code, data, and trained models for scientific reproducibility, we deem the disclosure of “the algorithm” itself (specifically, the mathematical underpinnings of the ML model) to be of no use. In fact, even knowing every single one of the hundreds of millions of parameter values in a given ML model would fail to provide even a spec of practically useful insight into the inner workings of a trained model. While we could re-create every decision, we would have no additional way to investigate its quality—much less assess its real-world impact. The disclosure of detailed information regarding generalization, model scope, and risk profiles, however, ensures utmost transparency.

### Future goals

#### Automatized ML development and optimization

The success of ML model development in practice crucially relies on machine learning experts to preprocess data (including e.g., imputation and cleaning), select and construct appropriate features (feature engineering; often with the help of domain experts), select an appropriate model family (e.g., neural networks or random forests etc.), optimize model hyperparameters (e.g., learning rate), and evaluate the model with regard to generalization, model scope, and risk profiles (including incremental utility, fairness, and security as outlined above). The complexity of this generic ML development pipeline, as well as the necessity to avoid data leakage and ensure proper evaluation can be challenging even for experienced researchers. Indeed, many studies forego optimizing the ML model development pipeline entirely. Indeed Arbabshirani et al.^48^ showed in a recent review of ML studies in the area of neuroimaging that 73% of studies employed a single machine (namely the Support Vector Machine) and almost no study optimized hyperparameters even for that single machine. This is particularly disconcerting as the *No Free Lunch Theorem*^49^ in ML clearly indicates that, without further knowledge of the process generating our data, no ML algorithm will, overall, perform better than any other. Even without any mathematical considerations, it is quite obvious that employing a single machine makes it extremely likely that another ML pipeline, algorithm, or setting thereof might have performed better. However, it may be possible that there are re-occurring data structures for which particular algorithms, pipelines, and hyperparameter values are optimal (e.g., default SVM parameters for variance normalized neuroimaging datasets^27^).

Against this background, we see increasing efforts to automatize the entire ML development process, from data preprocessing and feature engineering to model selection and hyperparameter optimization. Examples for this include auto-sklearn^50^ (a package focused on Bayesian hyperparameter optimization and machine selection), PHOTON (www.photon-ai.com; an ML framework enabling cross-platform ML pipeline construction, optimization, and evaluation), and Auto Keras^51^ (an open-source package based on TensorFlow for neural network architecture search). We expect this trend to accelerate in the years to come, automatizing most if not all ML model development steps. This, however, will increase the need for proper model evaluation and full *AI Transparency* as outlined above.

#### Learning complex models from small samples

While exceptionally successful in many areas, ML model training may require large amounts of data as models comprise at least as many free parameters as there are variables measured^52^. For complex models (e.g., Deep Learning), the number of parameters may easily increase to tens of millions of parameters^53^. Training such large models with “only” hundreds or even thousands of samples may induce so-called overfitting—a situation in which the large number of free parameters allows the model to essentially “memorize” all of the training data, leading to perfect performance on the training set, but extremely bad generalization to new, real-world data. While generally effective, the numerous countermeasures employed lower the complexity of what a model can learn, potentially rendering it unable to capture true associations in the data^54–56^. Thus, model performance is severely limited by the number of patients available, especially whenever high dimensional data sources such as neuroimaging data are of interest (commonly known as large P small N problems)^48^. Acquiring hundreds of thousands of patient samples, however, is usually not feasible—especially in psychiatry where comprehensive phenotype data is often crucial.

**Fig. 3:**
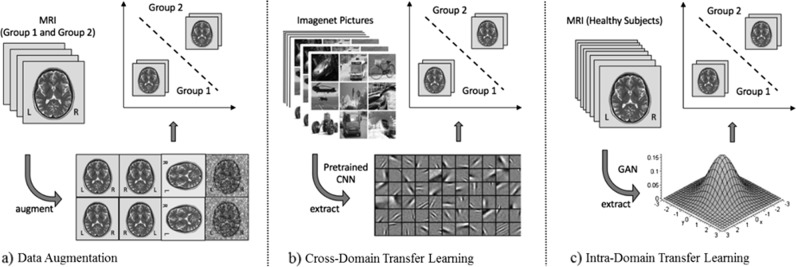
Illustration of workflows for the different techniques exemplified using Magnetic Resonance Imaging (MRI) data. **a** Data augmentation approach using stochastic and image processing methodology. **b** Cross-domain Transfer Learning applying low-level filters learnt by a Convolutional Neural Network (CNN) from the Imagenet database. **c** Intra-domain Transfer Learning deriving a statistical embedding from a large database of MRI images employing a Generative Adversarial Network (GAN)

The fundamental problem of model training on small datasets has recently been addressed in other areas with great success: First, using data augmentation—i.e., perturbing existing data to create new samples—as a mathematically principled means to artificially enlarge training set size, enables the generation of an arbitrarily large number of training samples using stochastic and image processing methodology custom-tailored for imaging data^57,58^ (including e.g., image synthesis, sample pairing techniques etc.; Fig. 3a). Second, transfer learning has been used to transform variables into a lower dimensional representation based on what has been learnt from other data sets^59–61^. In neuroimaging, for example, we could leverage a cross-domain transfer learning approach by extracting basic visual features of a pre-trained image classification algorithm (i.e., a Convolutional Neural Network, CNN) trained on 1.2 million natural images (Imagenet^57^, Fig. 3b). In the same vein, employing intra-domain transfer learning, we can extract general statistical properties from large datasets of healthy controls using state-of-the-art Generative Adversarial Neural Networks (GAN, Fig. 3c). Such GANs can, for example, represent the MRI data on a lower-dimensional manifold and enable the generation of an arbitrary number of MRI images from this distribution. While highly effective, these techniques have thus far not been systematically applied in psychiatry.

#### From ML models to clinical decision support systems

At current, applied ML in psychiatry shows promise yet is still in its early days. Once best practice is attained and proof-of-concept studies have been conducted, forward testing will be necessary to demonstrate prognostic stability, incremental utility, and real-world estimates of performance in the same context as they will be clinically deployed. In such a forward test, a clinician could make their prediction and clinical decision (e.g., will a patient enter remission if they prescribe a certain drug? If so, prescribe drug). In parallel, a trained model could also make its prediction, with both the model and clinician assessed at a 12-week endpoint. However, given the interpersonal nature of psychiatric care, it is unlikely that even if ML models prove to outperform clinician prognostication, they will ever solely drive the clinical decision-making process. Therefore, the testing of AI decision support systems alongside clinicians will likely provide a more realistic approximation of what to expect in terms of socially accepted clinical use. In this third study arm, a clinician could make a prediction and decision not only based off of their own clinical expertize, but the binary prediction of an ML model, as well as its predicted probabilities^62^. Here, the synergy between a clinician and an AI decision support system could be measured. In this case, not only the binary prediction but the probabilistic estimates are of importance. Therefore, model calibration should also be carefully considered (see Niculescu-Mizil & R. Caruana^63^ and the sklearn documentation^64^). If the collaboration of the clinician and the AI system significantly outperform clinician prognostication alone, the model could then move towards clinical deployment.

#### Psychiatry and beyond

Whilst the current overview has focused on the application of ML to psychiatric phenotypes, these recommendations will generalize to most areas of applied ML in medicine. However, as psychiatric disorders are defined based on deviations of phenotypic characteristics—not causal biological models—a disorder can be highly heterogeneous with regard to its biological underpinnings. Therefore, diagnostic labels in psychiatry likely contain noise not seen in other medical disciplines. This presents a set of unique challenges that are distinct to psychiatry, with implications for model selection, accuracy, reliability, reproducibility, and ceiling effects on model performance. In areas that do not face these challenges, for example, oncology, specific problems discussed, e.g., sample sized based systematic overestimation and overfitting risks may be less prevalent. Therefore, domain application should always be considered. Moving forward, only by parsing this heterogeneity in psychiatric phenotypes will we be able to lay the groundwork for more targeted models and improved patient care^65,66^.

## Conclusion

In this overview, we have suggested and discussed best-practice guidelines with the intention that they may help stakeholders, journals, and funding agencies to obtain a more realistic view of (1) what can be expected of a planned research project in terms of clinical utility, (2) how much closer a particular finding has brought us to clinical application, and (3) what remains to be done before we can expect improvements in daily practice. In addition, understanding how to develop, train, and evaluate ML models and publications might help researchers new to the field of ML in psychiatry to better plan and monitor their ML projects, creating robust best-practice procedures in the mid-term. In the long-run, we hope that these guidelines can help to channel funding, as well as media attention towards the most promising developments regarding improved patient care, circumventing the evident dangers of the current hype around machine learning and artificial intelligence.
